# Geometric regularity in webs of non‐orb‐weaving spiders

**DOI:** 10.1002/ece3.9839

**Published:** 2023-03-16

**Authors:** Martín J. Ramírez, Jonas O. Wolff, Peter Jäger, Martina Pavlek, Abel Pérez‐González, Ivan Magalhaes, Peter Michalik

**Affiliations:** ^1^ Museo Argentino de Ciencias Naturales “Bernardino Rivadavia” Consejo Nacional de Investigaciones Científicas y Técnicas Buenos Aires Argentina; ^2^ Zoological Institute and Museum University of Greifswald Greifswald Germany; ^3^ Arachnology, Senckenberg Research Institute Frankfurt am Main Germany; ^4^ Ruđer Bošković Institute Zagreb Croatia; ^5^ Croatian Biospeleological Society Zagreb Croatia

**Keywords:** Araneae, Araneoidea, orb web, silk, spinnerets, Synspermiata

## Abstract

Geometric regularity of spider webs has been intensively studied in orb‐weaving spiders, although it is not exclusive of orb weavers. Here, we document the geometrically regular, repetitive elements in the webs of the non‐orb‐weaving groups Leptonetidae and Telemidae for the first time. Similar to orb weavers, we found areas with regularly spaced parallel lines in the webs of *Calileptoneta helferi*, *Sulcia* sp., and cf. *Pinelema* sp. Furthermore, we provide a detailed account of the regular webs of *Ochyrocera* (Ochyroceratidae). The sections of the web with regularly disposed parallel lines are built as U‐shaped modules reminiscent of orb webs. It has been suggested that the regularly spaced parallel lines in the webs of Ochyroceratidae and Psilodercidae may be produced in a single sweep of their posterior lateral spinnerets, which have regularly spaced aciniform gland spigots, perhaps involving expansion of the spinnerets. To test this hypothesis, we compared the spacing between parallel lines with the spacing between spigots, searched for expansible membranes in the spinnerets, and examined the junctions of regularly spaced lines. The distance between parallel lines was 10–20 times the distance between spigots, and we found no expansible membranes, and the intersection of parallel lines are cemented, which opposes the single sweep hypothesis. Furthermore, we found cues of viscid silk in the parallel lines of the psilodercid *Althepus* and broadened piriform gland spigots that may be responsible of its production. Finally, we evaluated the presence or absence of geometrically regular web elements across the spider tree of life. We found reports of regular webs in 31 spider families, including 20 families that are not orb weavers and hypothesize that the two basic aspects of regularity (parallel lines spaced at regular intervals, and radial lines spaced at regular angles) probably appeared many times in the evolution of spiders.

## Graphical abstract

Geometric regularity of spider webs has been intensively studied in orb weaving spiders, although it is not exclusive of orb weavers. Here, we document the geometrically regular, repetitive elements in the webs of non‐orb weavers. Similar to orb weavers, we found areas with regularly spaced parallel lines as well as radial lines spaced at regular angles suggesting that such regular elements appeared many times in the evolution of spiders.

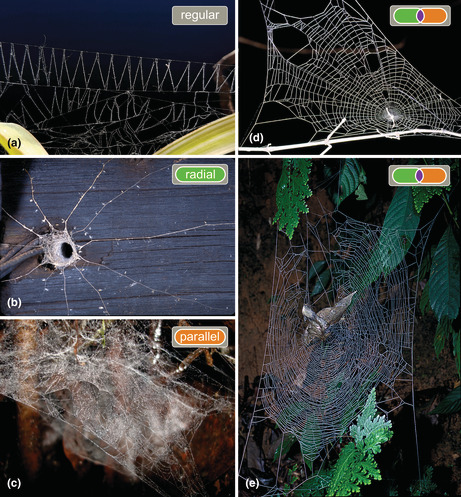

## 1 INTRODUCTION

Spider webs include some of the most complex and diverse examples of animal constructions, including orb webs, sophisticated trapdoors, diving bells and extensible droplets to fling at prey, among many other designs (Eberhard, 2020; Foelix, 2011). For many reasons, the extremely regular orb webs are the most intensively studied; the behaviors involved in their construction are highly stereotyped in sequence and choreography, with geometric elements that are readily identifiable (reviewed in Eberhard, 2020), and silk from different glands with varying physical properties used for specific threads (reviewed in Vollrath, 2000; Eberhard, 2020). Even though the general pattern is rather conserved, several versions of the orb web are described for six families in the clade Araneoidea, plus in Uloboridae and Deinopidae, with remarkable uniformity but also differences in structure and construction sequences. In addition to the final product, many of the constructional details, such as the sequence of assembly of the orb web and the cues used to guide construction routines, are shared across orb weavers (Coddington, 1986; Eberhard & Barrantes, 2015). Therefore, it is rather surprising that recent phylogenomic analyses did not obtain a monophyletic group of orb‐weaving spiders (e.g., Fernández et al., 2018; Garrison et al., 2016; Kallal et al., 2021) suggesting that this complex and stereotyped web‐building behavior evolved independently.

Geometrically regular webs are not unique to orb weavers. Further cases of repetitive web patterns have been reported in several other spider families (see Eberhard, 2020), but a comprehensive picture of the occurrence of regular web elements along the spider tree of life is still missing. This limits our understanding of how regular web structures may have evolved from irregular webs and if there are ancestral traits in construction behaviors shared by different lineages showing similar webs, such as in orb weavers.

Here, we document the geometrically regular, repetitive elements in the webs of Leptonetidae and Telemidae for the first time, two families that diverged early in the evolution of true spiders (Araneomorphae). Additionally, we explore the building process of regular webs in non‐orb weavers. Intrinsic to the geometrically regular designs is the ability to measure distances or angles, and, with this information, produce repetitive modules. Because spiders are essentially blind with respect to their webs, the measuring and subsequent attachment of new silk lines must depend either on tactile assessments such as tapping with legs and palps, the relative angles of legs, or on navigation (reviewed in Eberhard, 2020; see also Eberhard & Barrantes, 2015). An alternative mechanism, hypothesized by Hormiga et al. (2007) for some spiders now placed in the families Ochyroceratidae and Psilodercidae, suggests that the regularly spaced parallel lines may be produced in a single pass of their posterior lateral spinnerets, which have regularly spaced aciniform gland spigots (see also Eberhard, 2020). Such a mechanism would produce a geometrically regular design without the need of measuring. We tested this hypothesis using the indirect evidence of measurements of webs and spinnerets, the microstructure of the silk connections, and the morphology of the spinnerets of representatives of Ochyroceratidae and Psilodercidae.

## 2 MATERIALS AND METHODS

Fieldwork was conducted on several localities, as follows. *Calileptoneta helferi*: Two webs photographed, from USA, California, Del Norte Co., near Crescent City, N 41.7693° W 124.0120°, elev. 150 m, 25–29 June 2017, wet mixed forest with redwood, M. Ramírez & P. Michalik, no vouchers, identified from other specimens collected in same locality (MACN‐Ar 38,884, 38,945). *Sulcia* sp.: One web photogrphed, from Croatia: Špilja Crno jezero cave, Pelješac peninsula, Croatia, 17.63647° 42.84727°, elev. 417 m, 27 April 2019., 1 m, leg. & det. M. Pavlek, voucher CBSS AR5849. Telemidae, unidentified species (probably *Pinelema* sp.): One web photographed, from Laos, Khammouan Province, Thakek area, Ban Phôungam‐Mai, N 17°32.954', E 104°48.754', limestone cave Tham Payot, elev. ca. 180 m, 25 November 2012, photographed by P. Jäger; no voucher, tentatively identified from images. *Telema tenella*: One web photographed, from France, Pyrenees, La Forge, Cave near La Preste les Bains, 2.402722° 42.406667°, elev. 1135 m, 15 June 2019, 1 m, leg. & det. M. Pavlek, voucher CBSS AR5855. *Ochyrocera* sp. 1: Numerous webs photographed and sampled, from Colombia, Dept. Valle del Cauca, Mun. Yotoco, Reserva Nacional Forestal Bosque de Yotoco, near Buga, N3.87732° W76.43754°, elev. 1609 m, 1–4 December 2011, wet forest, M.J. Ramírez & M.A. Izquierdo (all vouchers ICN‐Ar 7516). Photographed webs: females 69–2, 69–9, 69–10, 69–13, 69–14, 69–15, 69–18, 69–19 (plus 27 collembolans Poduromorpha, probably Hypogastruridae); subadult male: 69–20; unidentified stages, no vouchers: Och‐G, Och‐H, Och‐K, Och‐L, Och‐M. Web samples: females 69–8, 69–11, 69–16 (all reconstructed in one night), 69–18; subadult male 69–20; males: 69–3, 69–21; unidentified stages, no vouchers: 69–17, Och‐M. *Ochyrocera* sp. 2: One web photographed, from Brasil, Rio Grande do Sul, Canela, Parque da Ferradura, between S29.265985° W50.843013° and S29.277867° W50.842911°, elev. 446–684 m, 28 January 2017, A. Pérez‐González, no voucher. *Althepus maechamensis*: One web photographed, one sampled, plus numerous webs observed, from Thailand, Chiang Mai, Doi Inthanon NP, cloud forest at summit, N18°35'21.4″ E98°29'10.2″, 4 October 2003, elev. 2590 m, ATOL Expedition. Immature with web photographed, voucher MACN‐Ar MR0360; immature web sampled, MACN‐Ar MJR‐1062; many adult males and females with webs examined, deposited in MACN‐Ar. An additional photograph, probably from the same species, provided by Hormiga et al. (2007: Figure 14d). Voucher specimens are deposited in Museo Argentino de Ciencias Naturales, Buenos Aires (MACN‐Ar), Croatian Biospeleological Society, Zagreb, Croatia (CBSS), Instituto de Ciencias Naturales, Universidad Nacional de Colombia, Bogotá (ICN‐Ar).

The webs were observed in the field using headlamps and photographed after powdering with cornstarch (Carico, 1977), if not already covered by the natural mist in high‐humidity environments. Web samples were taken with samplers as described in Ramírez et al. (2013) and observed in stereo, compound, or electron microscope. Several webs of *Ochyrocera* sp. 1 were marked and partially destroyed, and observed later in the early stages of reconstruction using red light. Scanning electron microscope (SEM) images were obtained after coating with gold–palladium and examined with a FEI‐XL30 scanning electron microscope under high vacuum. Measurements of web structures and spinnerets were taken from images of the silk samples, taken with a Leitz stereomicroscope and calibrated with a graded standard, then counting pixels with Adobe Photoshop and calculating with a spreadsheet.

**FIGURE 1:**
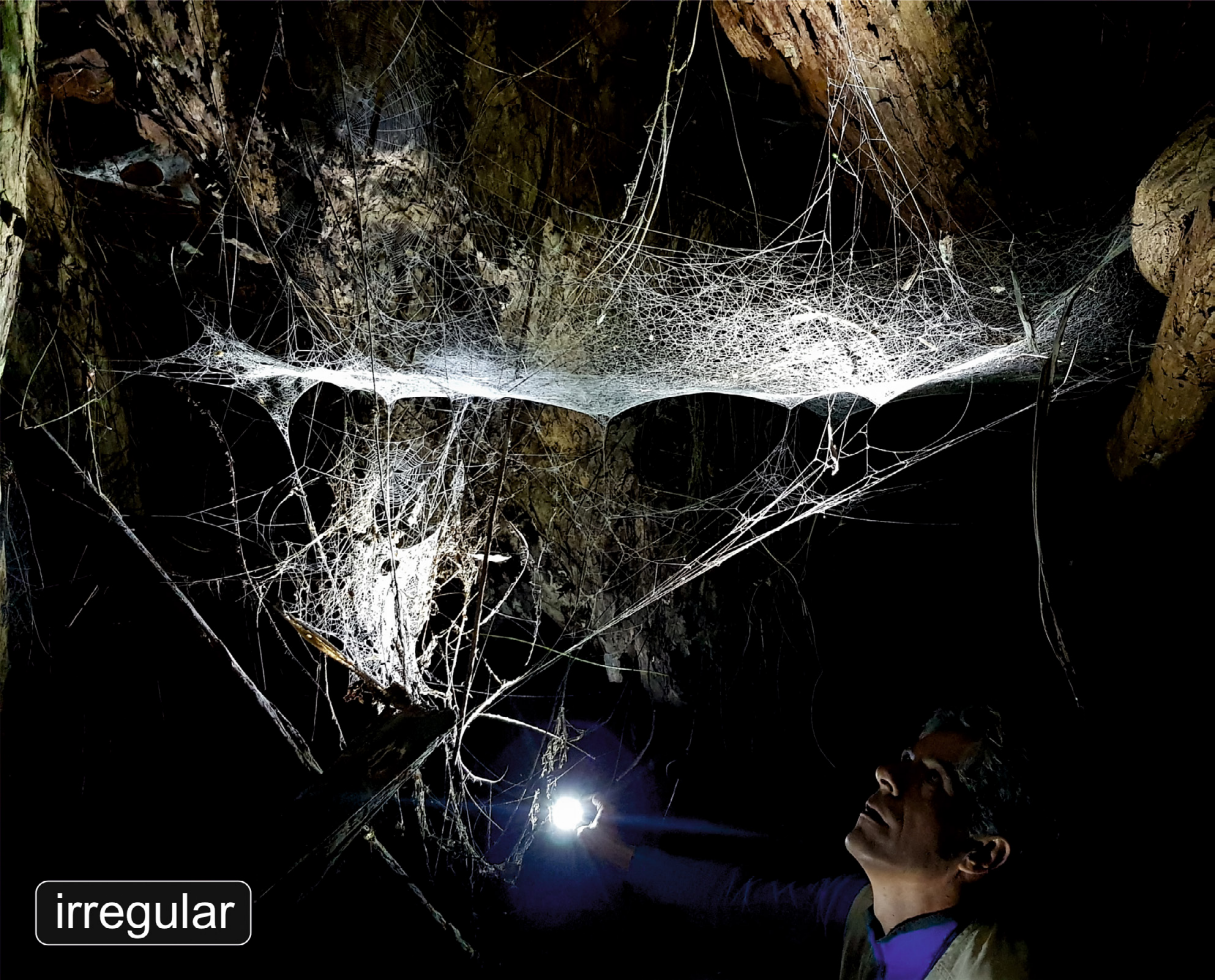
Example of a very large irregular web of a *Cambridgea* sp. (Desidae) from South Island, New Zealand.

**FIGURE 2:**
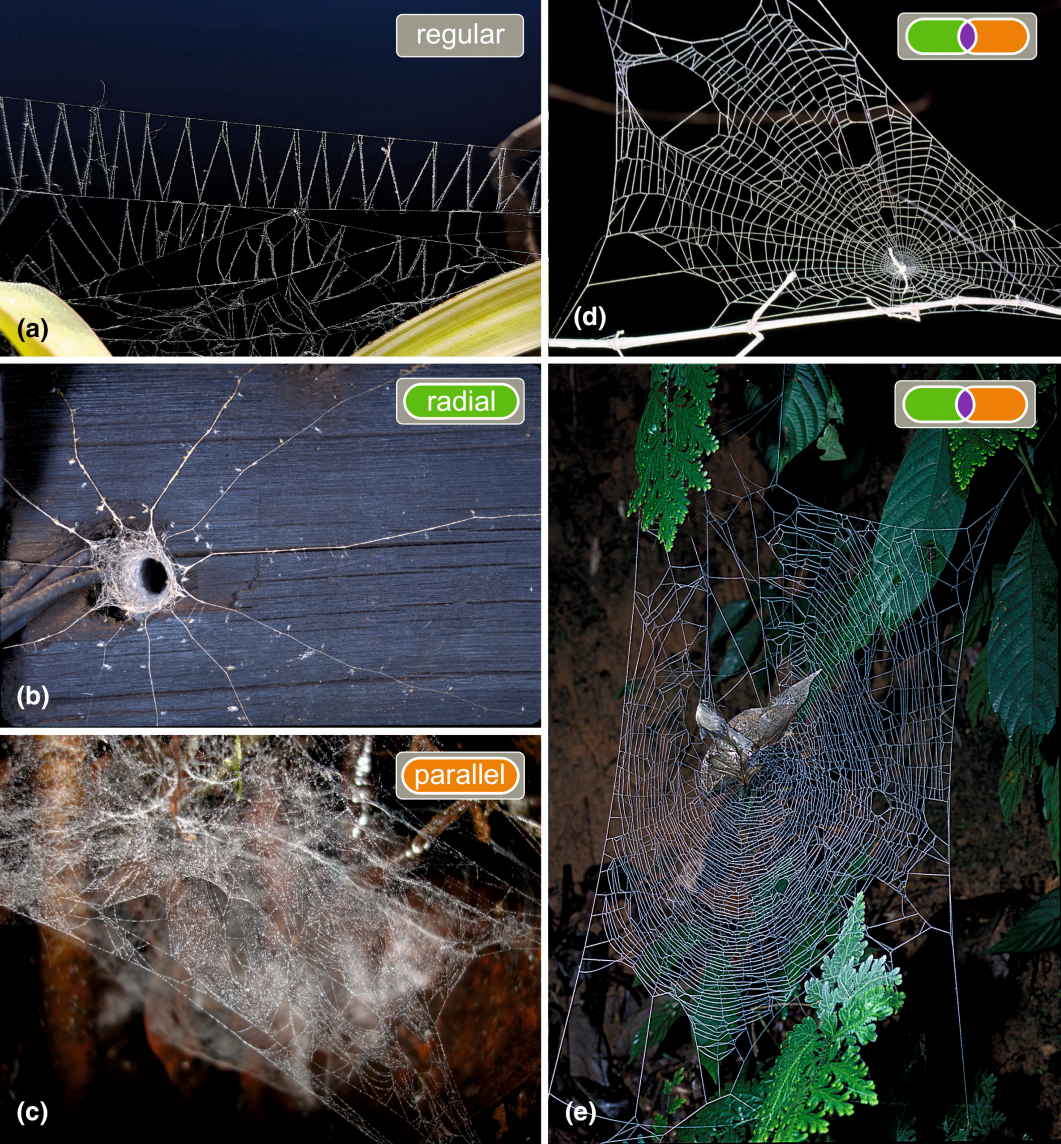
Examples of regular webs. (a) Regular web without parallel or radial regularity, of *Badumna longinqua* (Desidae) from Argentina. (b) Radial regularity in the web of *Ariadna* sp. (Segestriidae) from South Africa (photo Gustavo Hormiga). (c) Parallel regularity in the web of *Ochyrocera* sp. 1 (Ochyroceratidae) from Colombia. (d) Radial plus parallel regularity in an orb web of *Uloborus* sp. from South Africa. (e) Radial plus parallel regularity in a pseudo‐orb web of *Fecenia* sp. (Psechridae) from Thailand (photo Gustavo Hormiga).

Webs were categorized in irregular (Figure 1) and regular, this last with two sub‐categories of parallel and radial regularity (Figure 2; see also references and notes in Table S1). Regular webs in general were defined as those having repetitive web elements (modules) that consist of a stereotypic geometric arrangement of lines. “Parallel” regularity describes the highly parallel, similarly spaced layout of threads across several attachment points; this includes, for example, the webs of *Ochyrocera* (Figure 2c) and the rectangular mesh patterns of porteriines (Morrill et al., in press). “Radial” regularity refers to the repetitive regular angular separation of radii; this, for example, includes the sensing lines of some *Ariadna* species (Figure 2b) and some trapdoor spiders (Xu et al., 2015). Some regular webs have both radial and parallel regularity, as, for example, the araneoid and uloborid orb webs (Figure 2d), the pseudo‐orb webs of *Fecenia* (Figure 2e), and the web of *Oecobius* (Solano‐Brenes et al., 2022). Finally, there are some regular webs that do not have a parallel or radial regularity, such as the ziz‐zags or ladders made by some cribellate spiders (Figure 2a). Because of the intrinsic difficulty to characterize geometric regularity and the vast variability of spiders' webs, this summary must be taken as a coarse approximation. A more accurate definition of “geometric regularity” would be in a continuous scale and based on measurements; that strategy would require an extensive data acquisition. As an exploratory proxy, we instead used a loose qualitative threshold to include cases with moderate regularity (e.g., radial lines of *Kukulcania*, ladders of some Dictynidae, radii and spirals of *Oecobius*).

To summarize the distribution of webs with geometric regularity across the spider tree of life, a representative list of well‐documented spider species representing lineages with regular webs was compiled (Table S1), including their closer relatives with irregular webs; the list is based on (but not limited to) the extensive review of Eberhard (2020). Lineages with web building members were represented by one or two well‐known species. The summary is focused on non‐orb‐weaving spiders, and thus did not attempt to represent the multiple clades that presumably lost their orb webs within the predominantly orb‐weaving families Araneidae, Tetragnathidae, Mysmenidae, Symphytognathidae, Anapidae, and Uloboridae (see Eberhard, 2018). The selected species were scored for “general,” “parallel,” and “radial” regularity (Table S1). The more precisely defined subcategories “parallel” and “radial” were submitted to a phylogenetic analysis.

The inference of the phylogeny of the representative species follows the strategy of Wolff et al. (2022), using sequences of six molecular markers (mainly from Wheeler et al., 2017; see Table S1) and topological constraints for clades supported in phylogenomic studies (Kallal et al., 2021; Kulkarni et al., 2021; Ramírez et al., 2020; see Figure S1). The maximum likelihood consensus was obtained with IQ‐TREE 2.2.0, together with a sample of bootstrap pseudo‐replicates to account for phylogenetic uncertainty. These trees were dated with secondary calibrations (Table S2) from Magalhaes et al. (2020) using treePL (Smith & O'Meara, 2012). The phylogenetic mapping was made using the R packages phytools (Revell, 2012), geiger (Pennell et al., 2014), ape (Paradis et al., 2004) and sensiPhy (Paterno et al., 2018). Two models of discrete character evolution (ER, equal rates; and ARD, all rates different) were tested with Akaike information criterion using the consensus tree. The preferred model was subsequently used to produce 100 stochastic character mappings on each of 100 bootstrap trees. The average number of gains and losses was inferred from the stochastic mappings (100 for the consensus, 10,000 for the bootstrapped trees). For the phylogenetic mapping under parsimony, we used TNT (Goloboff & Catalano, 2016). We estimated phylogenetic signal under maximum likelihood using the δ statistic for discrete traits (Borges et al., 2019), and the consistency (ci) and retention (ri) indices under parsimony. (See Supplement for details of the phylogenetic analysis.)

## 3 RESULTS

All the studied species, except for *Althepus maechamensis* (Psilodercidae), are very small (few millimeters), and the lines in their webs are very thin and form superimposed layers.

### 3.1 *Calileptoneta helferi* (Leptonetidae)

**FIGURE 3:**
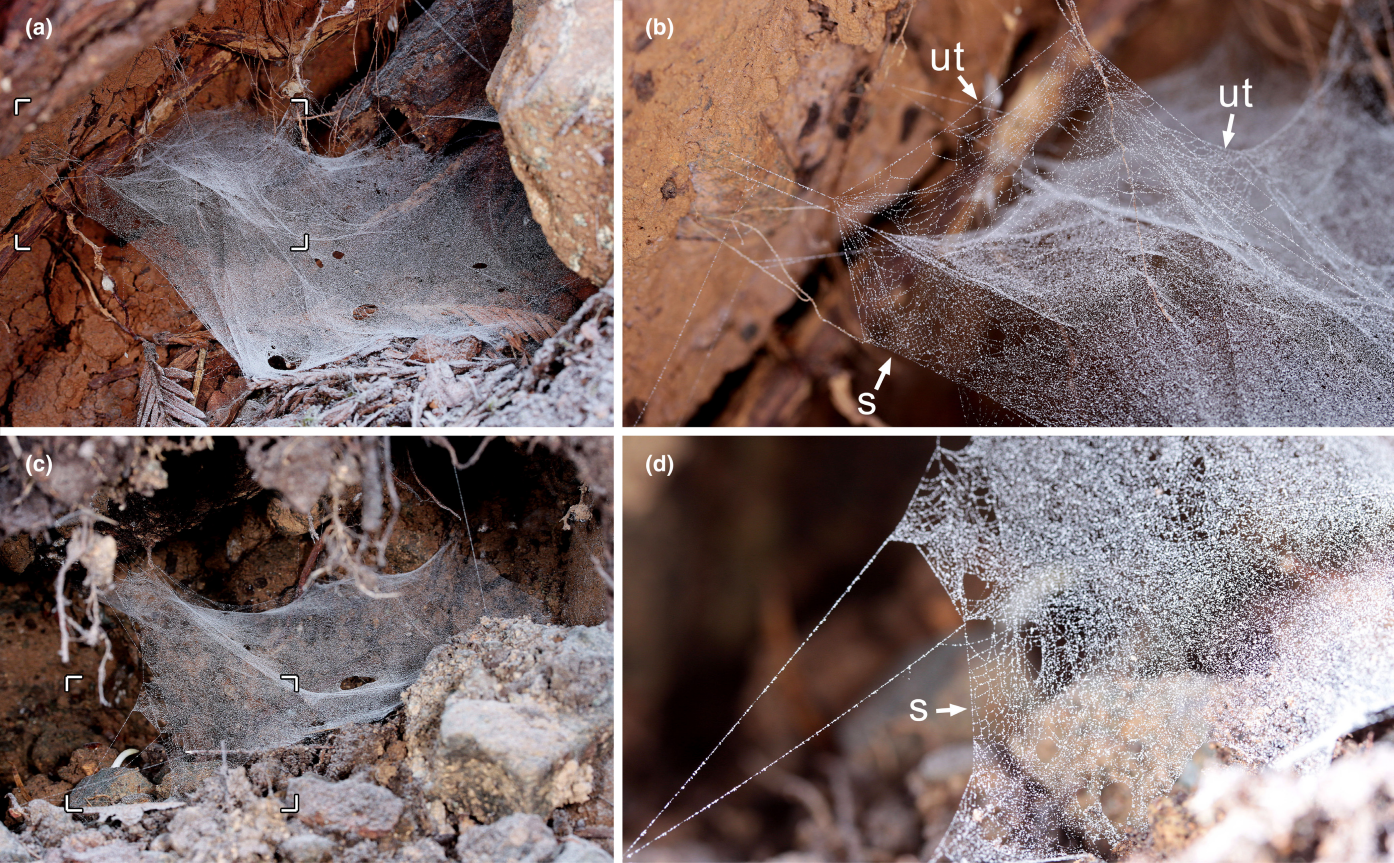
Webs of *Calileptoneta helferi* (Leptonetidae). (a) complete web with sparse upper tangle and some superimposed layers in the sheet. (b) detail of sector marked in (a), arrows to regular filling with parallel lines in the sheet (s) and upper tangle (ut). (c) complete web, with no superimposing layers in the sheet and no upper tangle. (d) detail of sector marked in (b), showing regular filling in the sheet(s).

The web is a nearly horizontal sheet or sheets (Figure 3). Of the two observed webs, one had a sparse upper tangle and some superposition of layers in the sheet (Figure 3a), and the other had neither (Figure 3c). The spider hangs in inverted position from the sheet. The filling of the sheet is tight, except for some borders less densely covered, showing arrays of nearly parallel lines that crossed in several parts, forming a design of diamonds (Figure 3b,d). The geometric filling was visible in the main sheet and between lines of the upper tangle.

### 3.2 *Sulcia* sp. (Leptonetidae)

**FIGURE 4:**
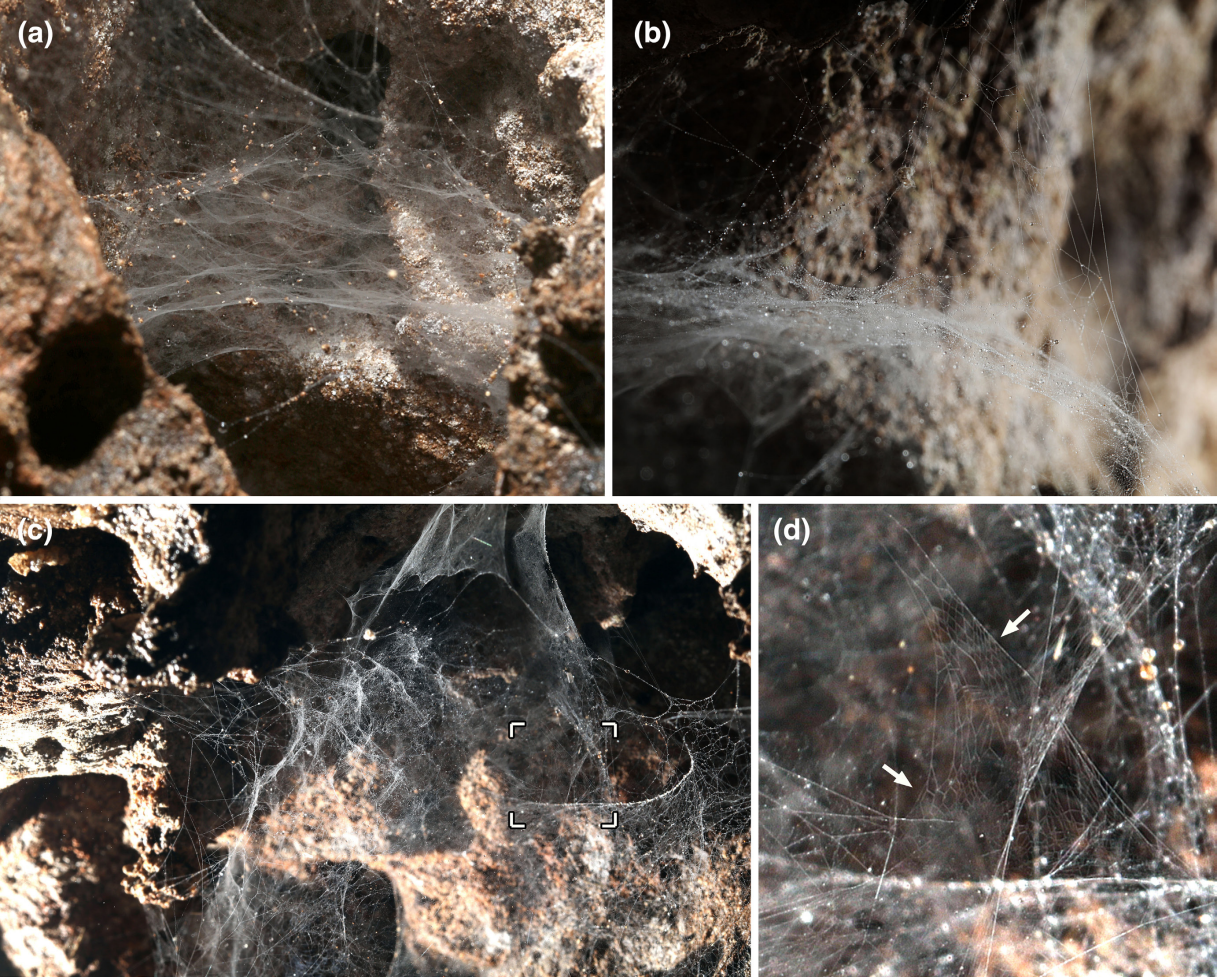
Webs of *Sulcia* sp. (Leptonetidae). (a) web taken from the side. (b) close‐up from the side. (c) web from above. (d) detail of sector marked in (c), with arrows pointing to areas with crossing parallel lines. Photos by Tin Rožman.

The web is an irregular structure, with many superimposed dense sheet‐like layers (Figure 4). The spiders hang in inverted position from the web. An area with sparse silk shows a regular pattern of two sets of parallel lines that intersect, forming diamonds (Figure 4d).

### 3.3 Telemidae, unidentified species (probably *Pinelema* sp.)

**FIGURE 5:**
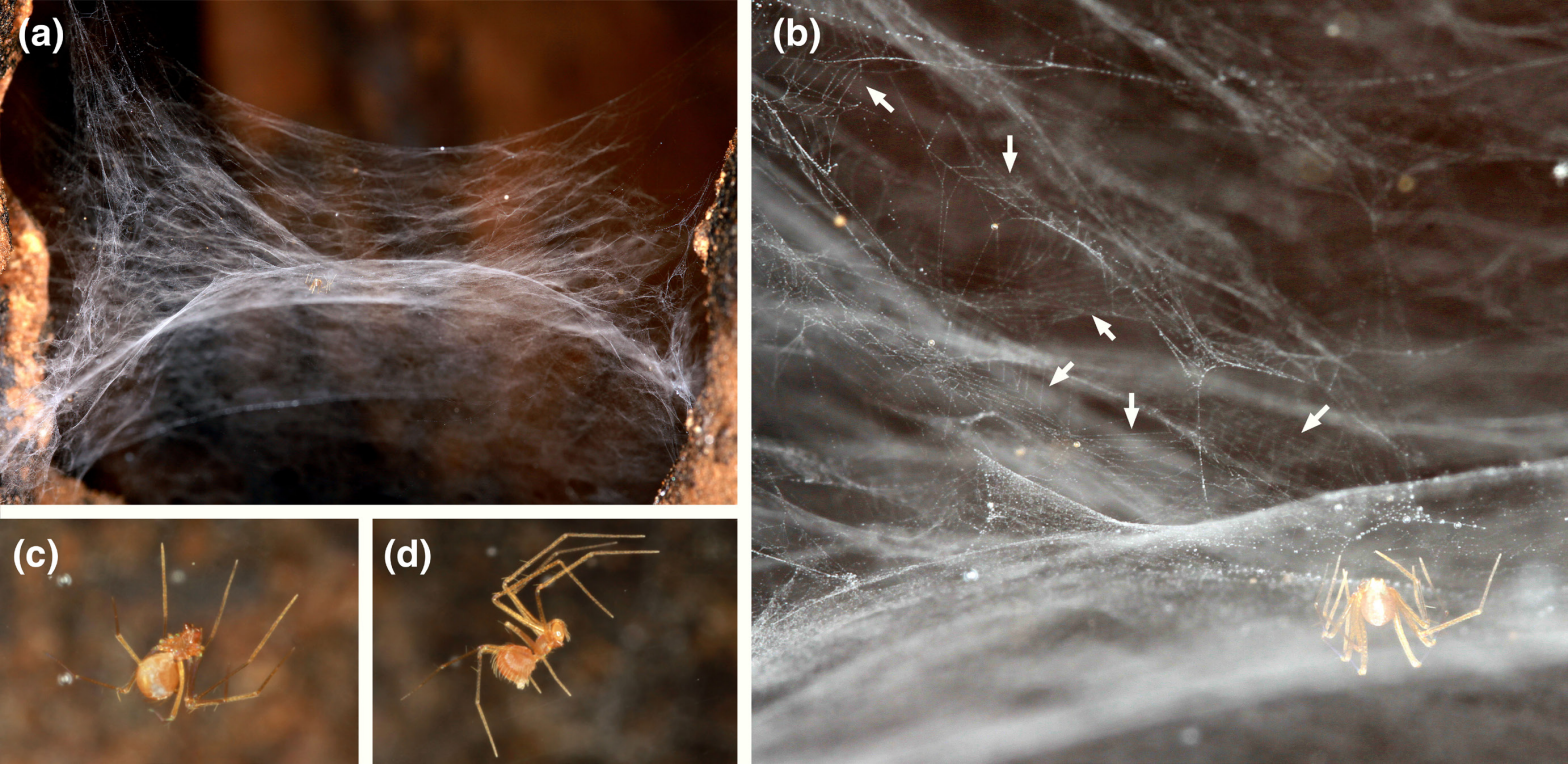
Web of a Telemidae, probably *Pinelema* sp. (a) web taken from the side. (b) detail with arrows to areas with regularly spaced lines. (c) close‐up of a female. (d) close‐up of a male.

The web has a domed sheet, with many smaller sheet layers above the dome (Figure 5a,b). The spider hangs from the sheet in inverted position (Figure 5b,c), one male was observed to walk on the upper sheet side (Figure 5d). Many areas above the dome are regular with parallel lines (Figure 5b).

### 3.4 *Telema tenella* (Telemidae)

**FIGURE 6:**
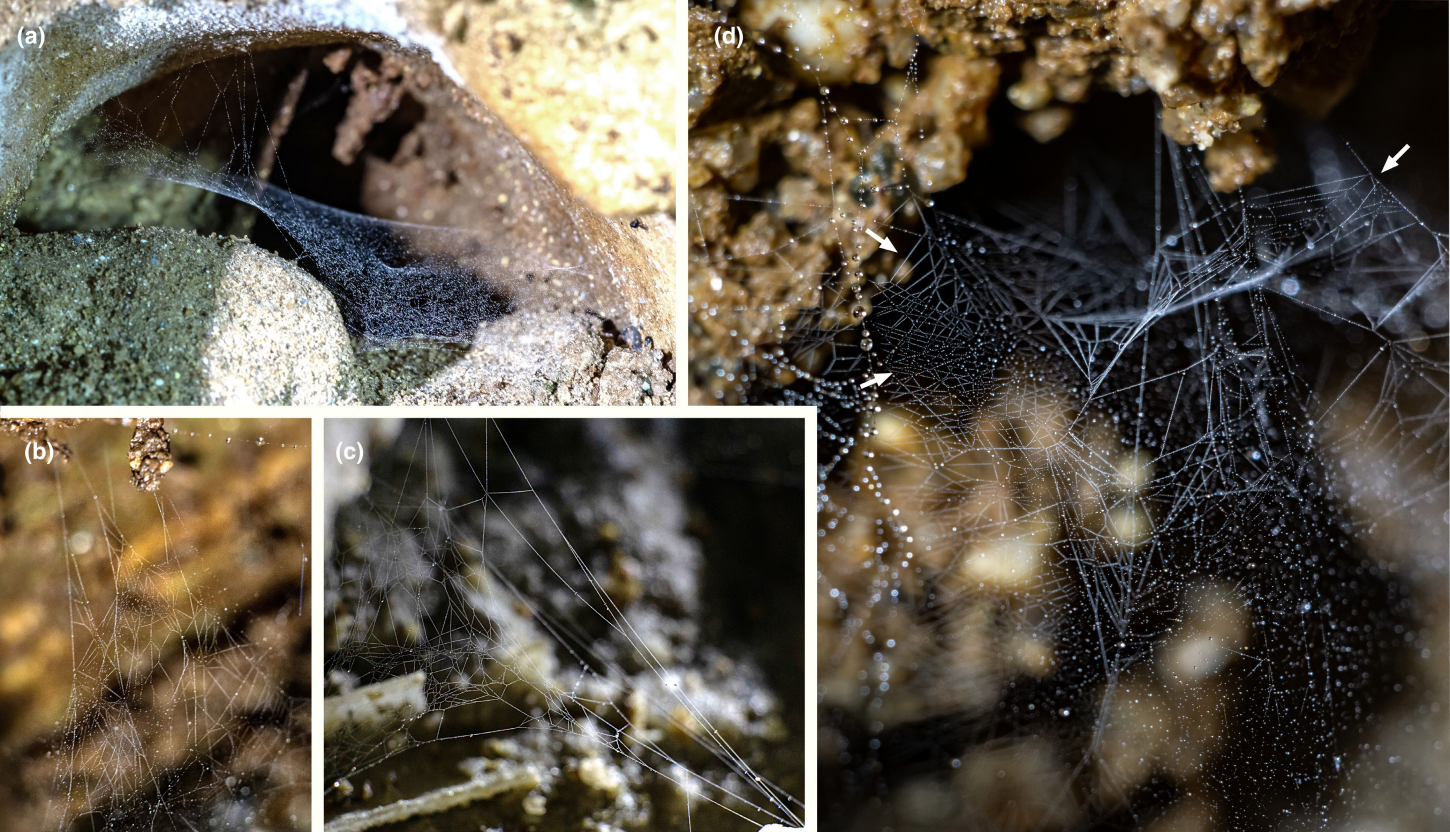
Webs of *Telema tenella* (Telemidae). (a) entire web. (b–d) details of sectors in other webs. Arrows in (d) point to areas with evenly spaced parallel lines. Photos by Tin Rožman.

The web is a sheet, with only few supporting lines from above (Figure 6). The spider hangs from the sheet in an inverted position. Some areas of the sheet and between the supporting lines were filled by equally spaced parallel lines (Figure 6d). In other areas of the sheet, no parallel lines were visible (Figure 6b,c).

### 3.5 *Ochyrocera* sp. 1 (Ochyroceratidae)

**FIGURE 7:**
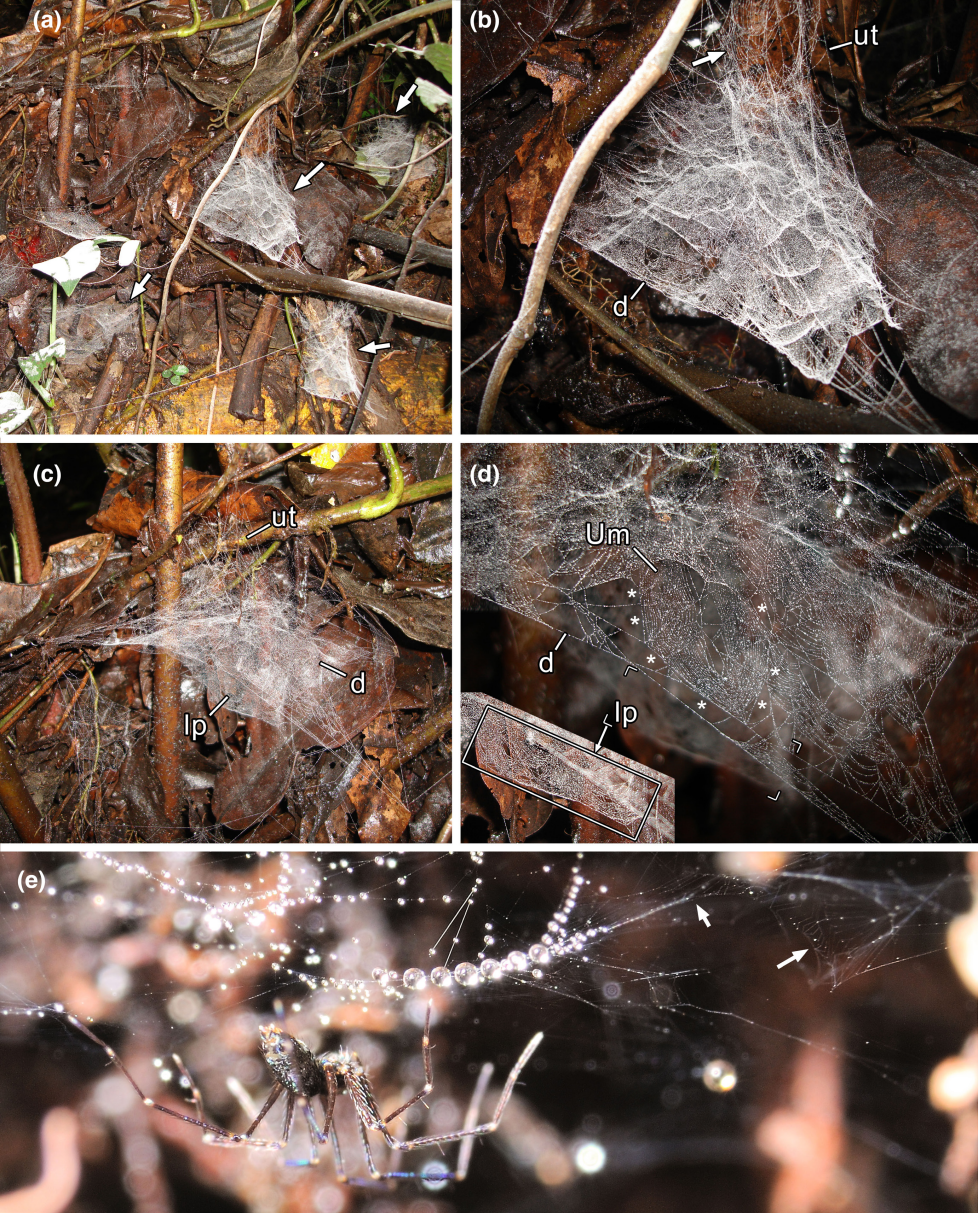
Webs undescribed *Ochyrocera* sp. 1 (Ochyroceratidae) from Colombia (a–d) and undescribed *Ochyrocera* sp. 2 from Brazil (e). (a) four intact webs in forest floor (arrows) (no voucher). (b) detail of central web; arrow indicates a sector of the upper tangle filled with parallel lines (no voucher). (c) intact web from adult female (voucher 69–10). (d) same, detail, showing a freshly spun U‐module in the dome (Um, encircled by *), and inset (bottom left) in lower focal plane, showing the lower platform; both are at approximately the same scale. (e) Female and web, arrows to sectors showing parallel lines. d, dome; lp, lower platform; Um, U‐modules; ut, upper tangle.

**FIGURE 8:**
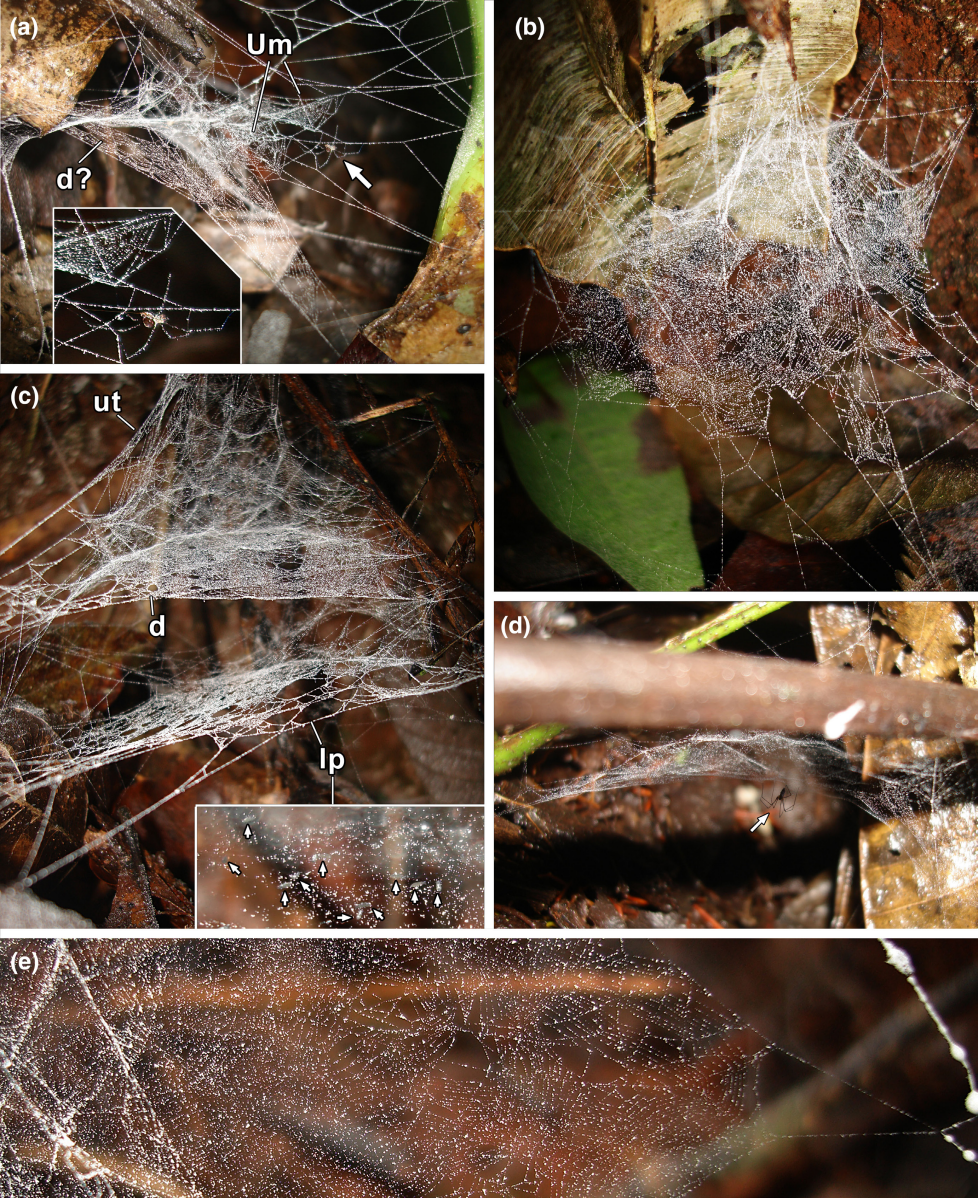
Webs of *Ochyrocera* sp. 1 (Ochyroceratidae), all rebuilt after destruction of the original webs. (a) recently initiated web from adult female, showing the first U‐modules on top, and a flat sheet below that will probably become the dome; arrow to spider, shown in inset (voucher 69–15). (b) freshly initiated web with a few modules, before the construction of the dome (Och K, no voucher). (c) advanced web from adult female, showing the lower platform; inset with arrows to collembolans on lower platform (voucher 69–19). (d) early completed web from adult female, missing the lower platform (arrow to spider) (voucher 69–13). (e) dome of a web rebuilt by a penultimate male, showing sectors of crossing parallel lines (voucher 69–20). (d), dome; lp, lower platform; Um, U‐modules; ut, upper tangle.

Some of the following observations were anticipated as personal communication from MJR in Eberhard (2020). The webs of *Ochyrocera* sp. 1 were abundant near the forest floor (Figure 7a) and on banks beside a trail. Webs are complex (Figure 7b,c) and were composed of (1) an upper tangle, usually attached to sticks or plants, (2) several partially superimposed U‐shaped modules made of curved parallel lines just below the tangle, (3) a dome, which also included U‐shaped modules (Figure 7d, asterisks), but in a continuous surface (where the spiders were usually hanging), and (4) a lower platform, made of a horizontal, continuous sheet tightly filled with patches of parallel lines (Figure 7d, inset). This platform is attached to the borders of the dome and to lateral attachments to the substrate (Figure 8c). The web is attached to the substrate by lines from the border of the dome and from the upper tangle.

We starched and partially destroyed several webs of *Ochyrocera* sp. 1 and returned later to observe the early stages of reconstruction (Figure 8). The spiders probably reused some of the attachment points of previous webs, but never incorporated any of the old lines, which were starched. The spiders first built a skeleton web, then produced U‐modules within, starting from the top and working downward (Figure 8a,b). The first U‐modules were about as wide as long, with outer portions of very regular parallel curving lines similar to what is found in orb webs (Figure 8a, inset, 8b). The U‐modules on the dome were usually more elongate (Figure 7d, center), sometimes crossing each other (Figure 8e), or straight bands (Figures 7d, bottom right; 8a). Some sectors of the upper tangle were filled with groups of parallel lines as well (Figure 7b). Our observations suggest that the general sequence of construction is from top to bottom, with the lower platform the last element in the construction; in support of this, Figure 8d shows a reconstructed web still missing the lower platform.

**FIGURE 9:**
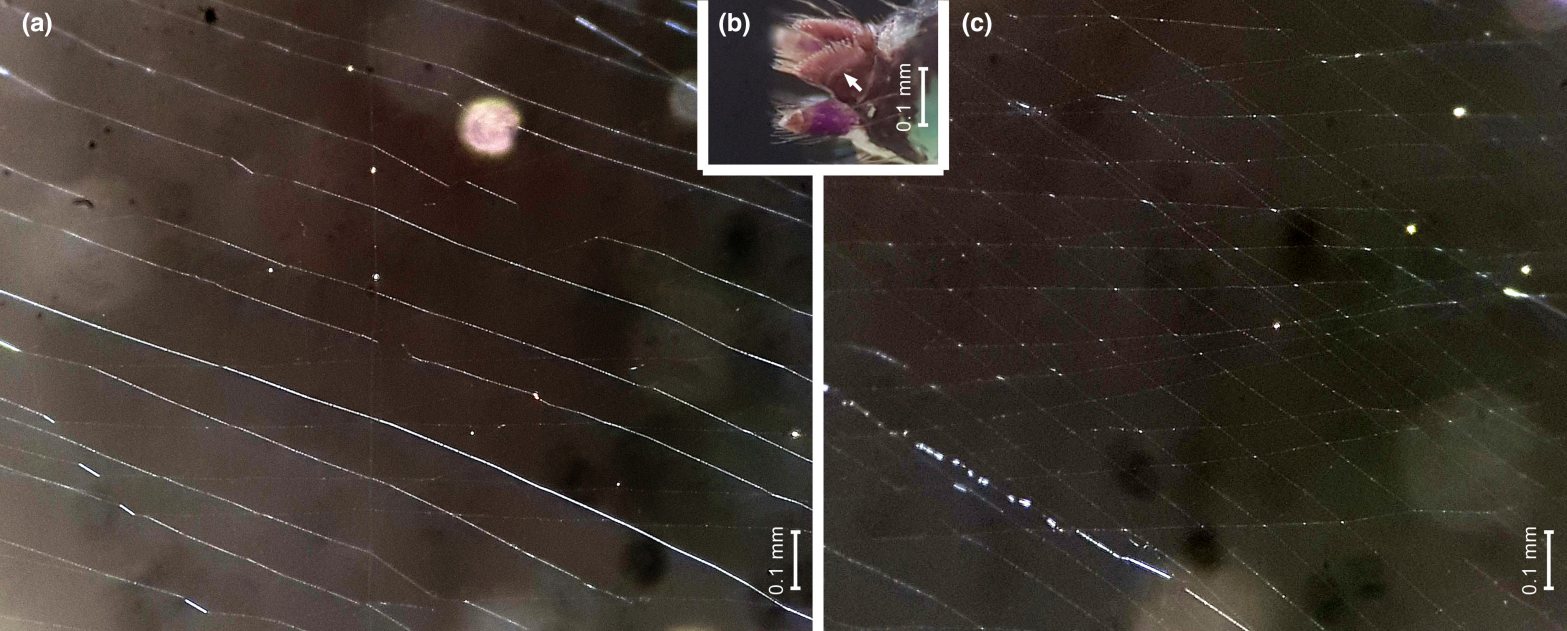
Samples of webs and spinnerets of adult female *Ochyrocera* sp. 1 (voucher 69–9); all images at the same scale. (a) sector with parallel lines. (b) detail of spinnerets, arrow to right PLS with row of aciniform gland spigots. (c) sector with two sets of parallel lines in the same plane.

Web samples of *Ochyrocera* sp. 1 were taken from several specimens, of which three had clear sectors of parallel lines that can be measured under the microscope (Figure 9a,c). The separation of parallel lines was measured in six sectors of these three webs (mean 0.106 mm, SD 0.037 mm, *n* = 46). Two voucher specimens were collected from the measured webs, of which one female (voucher 69–16) had good contrast, allowing to count 16 aciniform spigots in a line on each PLS (Figure 9b). The mean distance between each spigot was 0.0087 mm (total left PLS spigot field 0.131 mm). The separation between consecutive parallel lines in the web of that spider was, on average, 0.087 mm (*n* = 28), that is, 10 times larger than the separation between consecutive spigots.

### 3.6 *Ochyrocera* sp. 2 (Ochyroceratidae)

We provide a complementary image of a female on a web, with visible sectors of parallel lines (Figure 7e).

### 3.7 *Althepus maechamensis* (Psilodercidae)

**FIGURE 10:**
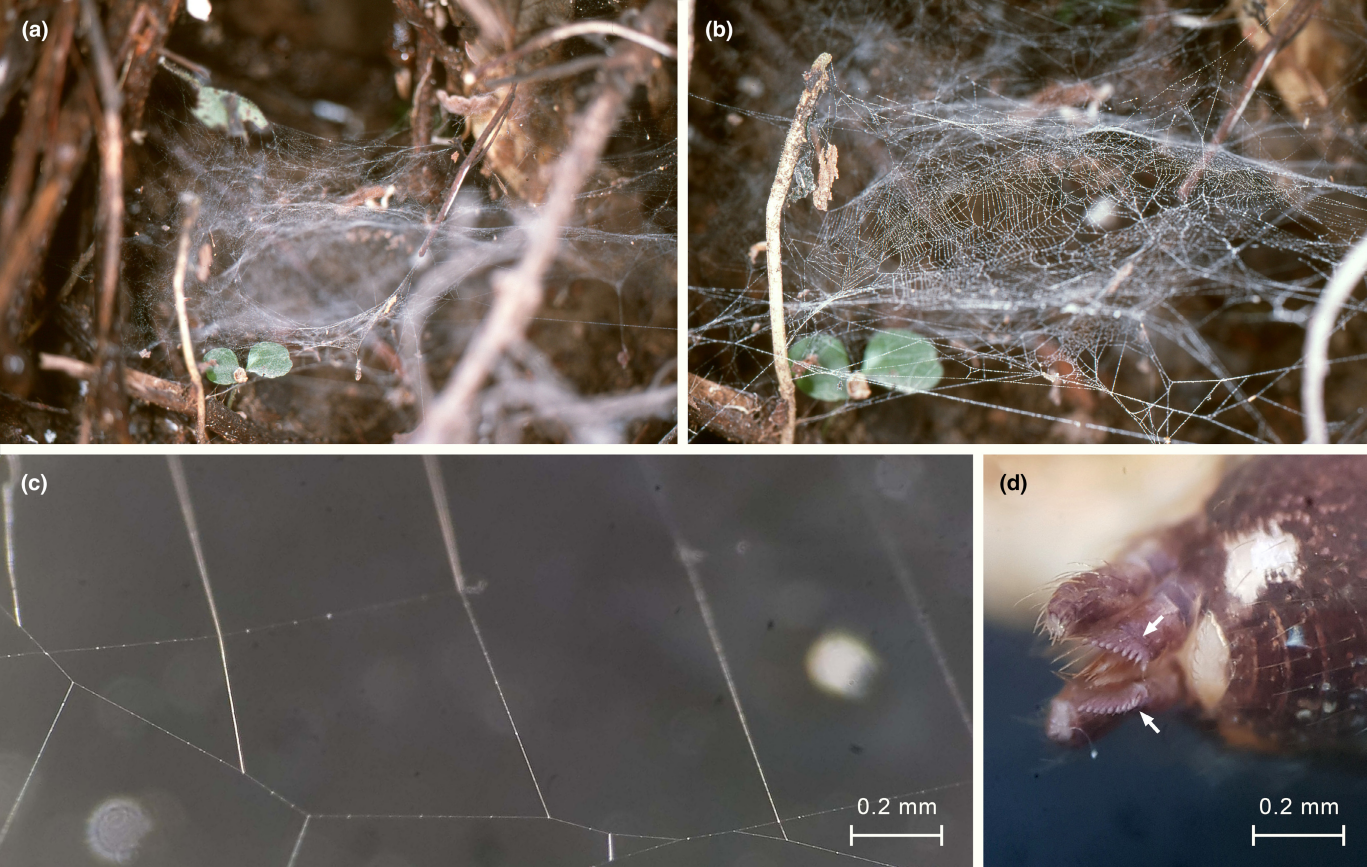
*Althepus stonei* (Psilodercidae). (a) web. (b) detail of domed sheet. (c) a sector of regularly disposed parallel lines, photographed with stereomicroscope (voucher MJR‐1062); same scale as (d). (d) spinnerets, dorsal lateral view, arrows to row of aciniform gland spigots on posterior lateral spinnerets (voucher MJR‐1062); same scale as (c).

**FIGURE 11:**
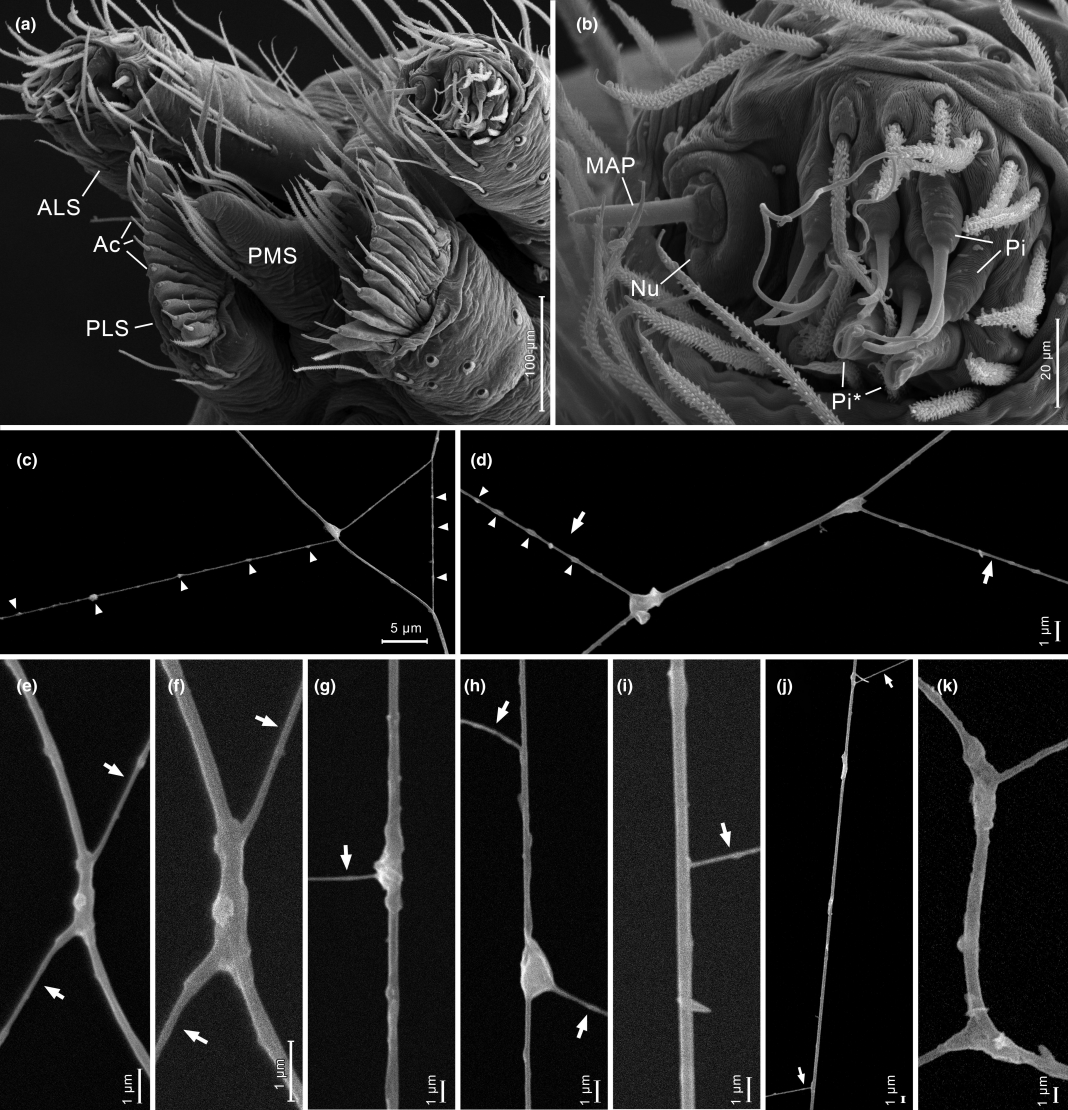
SEM images of *Althepus maechamensis* and its web. (a) male spinnerets. (b) male left anterior lateral spinneret. (c–k), details of silk threads and attachments; regularly spaced parallel lines are marked with arrows, probable droplets of viscid silk are marked with triangles. (c) silk lines with probable sticky droplets; the thinner line may be one of the regularly spaced parallel lines, but it was in an area with many crossing lines. (d) one of the regularly spaced parallel lines (arrows) with probable sticky droplets (triangles), and two glued attachments on a thicker line. (e–g) glued attachments of a parallel line (arrows) with a thicker line. (h) parallel line (arrows) with a glued attachment on a thicker line, plus a segment adhered without attachment. (i–j) parallel lines (arrows) adheed to thicker lines without attachments. (k) attachments between thicker lines in the web, without parallel lines involved. Ac, aciniform gland spigot; ALS, anterior lateral spinneret; MAP, major ampullate gland spigot; Nu, nubbin; Pi, piriform gland spigot; Pi*, broad piriform gland spigot; PLS, posterior lateral spinneret; PMS, posterior median spinneret.

A photographed web had a domed sheet with a tangle above. The sheet was composed of sectors of parallel lines, often crossing each other (Figure 10a,b, voucher MACN‐Ar MR0360; see also Hormiga et al., 2007: Figure 14d). Web samples were taken from another specimen (Figures 10c,d, and 11b–j) and examined under the microscope. While several areas of the web were adhered to the glass slide, other were suspended, especially near the elevated borders of the sticky tape of the slide. The separation of parallel lines was measured in four sectors (mean = 0.402 mm, SD = 0.075 mm, *n* = 14). The specimen collected from the measured web was immature (voucher MACN‐Ar MJR‐1062) and was identified by comparison with adult specimens from the same locality. Each posterior lateral spinneret (PLS) has nine aciniform gland spigots in a line, with a mean distance between each spigot of 0.019 mm (total left PLS spigot field 0.153 mm). The separation of parallel lines in the web is, on average, 21 times larger than that of the spigots. We examined the silk sample with SEM. The regularly spaced parallel lines were thinner than other lines in the web. Many of the intersections of the parallel lines with other lines of the web had a glue‐like mass of material (Figure 11d–h). In some intersections, the parallel lines were contacting or adhering without an evident mass of glue (Figure 11h top, i–j). Other lines in the web, not regularly spaced, were thicker and had glued junctions as well (Figure 11j). In several segments, the parallel lines had regularly spaced thickenings, compatible with droplets of viscid silk. The spinnerets of a male specimen examined with SEM (Figure 11a,b) had the following complement of spigots: anterior lateral spinneret with a single major ampullate gland spigot accompanied by a nubbin, nine piriform gland spigots with narrow shafts, and two piriform spigots with broad shaft; posterior median spinneret with a single spigot, identified as a minor ampullate gland spigot; posterior lateral spinneret with a row of 12 aciniform gland spigots, without traces of extensive folding membranes.

### 3.8 Phylogenetic distribution of regular webs

**FIGURE 12:**
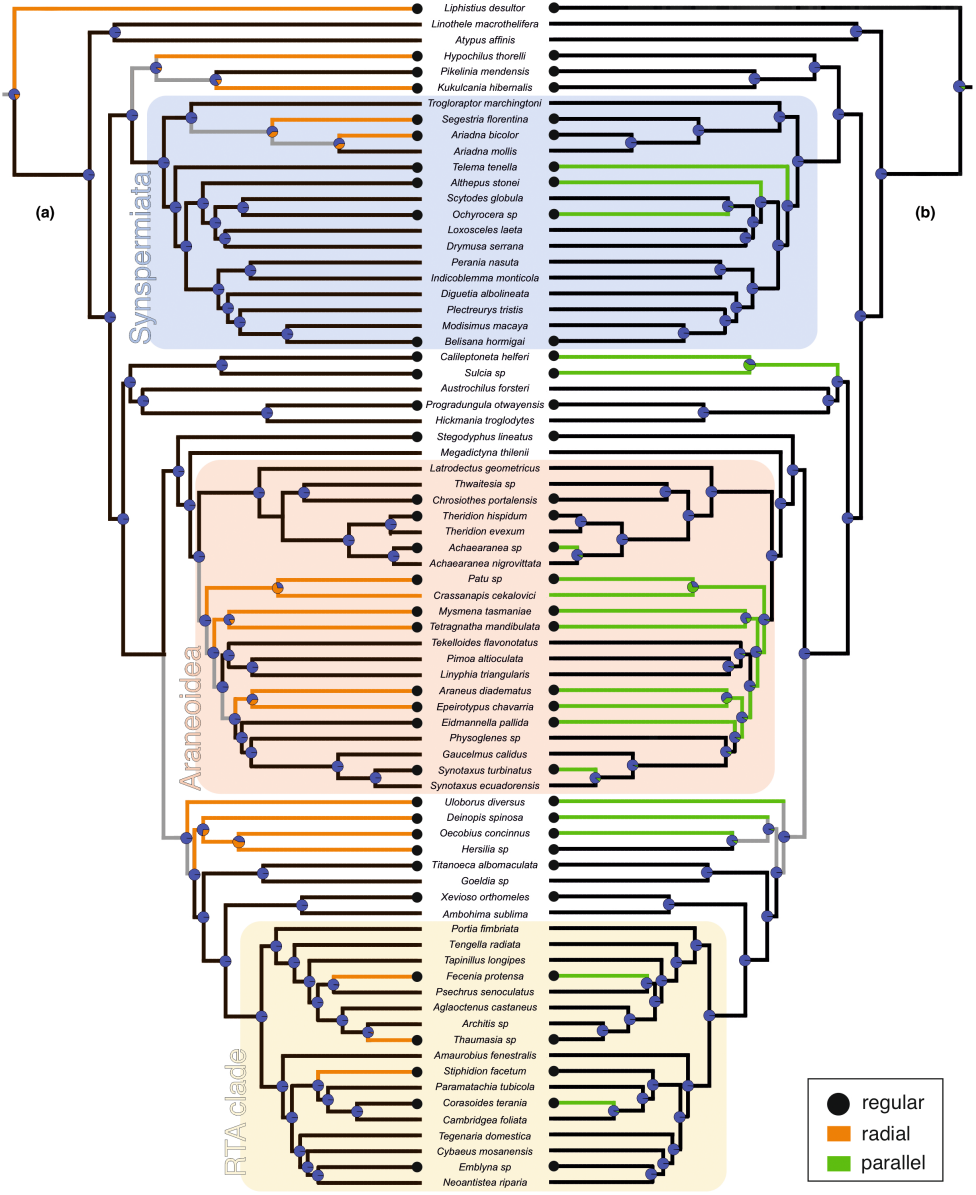
Geometrically regular webs on a summary phylogeny. Species with some kind of regularity in their webs are marked with a black circle, regardless of having or not parallel or radial regularity. Mapping on the branches produced with parsimony showing the occurrence of (a) radial regularity of lines equally spaced, and (b) parallel regularity of radii spaced by repetitive angles. The maximum likelihood estimations of trait probabilities are represented on the nodes.

We compiled a list of 37 species representative of lineages with geometrical elements in their webs (Table S1). These species belong to 31 families of Mesothelae and all major clades of Araneomorphae (Figure 12, black circles), including 20 families not affiliated with orb weavers. In order to provide a context of the occurrence of geometrical regular web elements, we added 38 species representing the main lineages with irregular webs, in between clades with regular webs. The mapping of radial regularity implied 13 steps, with 7–13 independent origins across the main clades of spiders and 0–6 losses (Figure 12a). The parsimony mapping of parallel regularity implied 14 steps, with 10–12 independent origins across the main clades of Araneomorphae and 2–4 losses (Figure 12b). The maximum likelihood and Bayesian estimations are largely coincident with the parsimony results in inferring multiple independent acquisitions of parallel and radial regularity. The stochastic mapping inferred a median of 20 gains and 4 losses of parallel regularity, and 18 gains and 3 losses of radial regularity (Table S3); the same results were reproduced when a sample of 100 bootstrapped trees were considered (Table S4). Although highly similar in implying multiple independent acquisitions, we preferred the mappings obtained with parsimony (Figure 12) because they are in better agreement with current ideas of web evolution; for example, in sister groups with regular webs, the ancestor is inferred regular as well (compare for example the sister groups Araneidae + Theridiosomatidae in Figure 12 and Figure S2). The phylogenetic signal of both characters is very low (parallel regularity, δ = 0.8029, ci = 0.0714, ri = 0.2778; radial regularity, δ = 0.8506, ci = 0.0769, ri = 0.2941).

## 4 DISCUSSION

We have documented here the occurrence of geometrically regular webs in telemids and leptonetids, which, by their disparate phylogenetic placement, are important to infer the origins of regularity in the design of spider webs. Ledford and Griswold (2010: 8) reported that leptonetids and telemids spin “a pattern of parallel lines of silk similar to that recorded for the Ochyroceratidae.” Our study confirms this observation by describing the regular patterns in the webs of the leptonetids *Calileptoneta helferi* and *Sulcia* sp., and the telemids *Telema tenella* and cf. *Pinelema* sp. How are these parallel lines produced? Hormiga et al. (2007) suggested that the parallel lines in ochyroceratids should be produced by the regularly spaced aciniform spigots in the posterior lateral spinnerets (PLS). It is remarkable that the spiders here reported making parallel lines belong to four spider families with an uncommon pattern of aligned spigots on the posterior spinnerets (Ledford & Griswold, 2010; Platnick et al., 1991). It is thus tempting to associate these parallel lines as produced in a single swath from the regularly spaced spigots, as Hormiga et al. (2007) did. Our measurements on the web and spinnerets of specimens of *Althepus maechamensis* and *Ochyrocera* sp. 1 revealed that the distance between parallel lines was 21 and 10 times the distance between spigots, respectively. Upon comments from one of us (MJR), Eberhard (2020: 52) suggested that the spinnerets could be somehow inflated to span a larger size. According to our measurements, the PLS of *Althepus maechamensis* should expand 20 times to match the separation of lines, which would span more than the body size of the spider; and the PLS of *Ochyrocera* sp. 1 should expand 10 times, which is the entire length of the opisthosoma (also compare the spider size and the separation of parallel lines in Figure 9a). Spiders of the family Gnaphosidae are known to inflate their anterior lateral spigots and spread widely their piriform gland spigots (Ramírez, 2014; Wolff et al., 2017, 2021), with the most extreme case in molycriines (Rodrigues & Rheims, 2020). The stretching of spider cuticle depends on accordion‐like folding of the cuticle, which becomes extended on stretching; such folds are evident in SEM images, as in the abdominal cuticle, that may stretch two‐fold upon feeding or egg development (Hackman & Goldberg, 1987). More drastic expansions are attained by greatly folded membranes that expand by hydrostatic pressure, as in the male copulatory organs (see Huber, 2004). The mechanism of spinneret expansion in gnaphosid spiders include long piriform spigots on flexible membranous areas, which are deeply invaginated in resting position (Wolff et al., 2017). Our examination of the spinnerets of *Althepus maechamensis* did not reveal any extensive system of folded membranes that a 20x expansion would require. The same is true for the spinnerets of other species of *Ochyrocera* previously examined (Platnick et al., 1991; Hormiga et al., 2007; Pérez‐González et al., 2016; Castanheira et al., 2019).

What could be the function of the regularly spaced aciniform spigots in the four families discussed above? Since these occur in immatures and adults of both sexes, they probably serve some general function. Many spiders (e.g., some araneids, tetragnathids, deinopids, uloborids, and eresids), have a brush of aciniform gland spigots in front of the posterior median spinnerets that is used for rapidly wrapping preys with a band of silk (see Coddington, 1989). It is thus possible that the spinnerets with aligned aciniforms can be operated to produce a wide band of aciniform silk from both pairs of posterior spinnerets at the same time, and thus provide an efficient wrapping mechanism.

Our SEM examination of regularly spaced silk lines of *Althepus maechamensis* revealed that many of the intersections of those lines with other lines in the web are cemented by a mass of silk, while others are dry. The cemented intersections are compatible with silk attachments, probably made with piriform silk, which spiders make one by one (see Greco et al., 2019; Wolff et al., 2021). In contrast, the brushing of spigots, including aciniforms, has been shown to produce silk intersections that are not glued by thick masses of silk (Eberhard & Hazzi, 2013, 2017). We consider that the order of magnitude between silk and spigot separation, the absence of a system of membranes that would allow the expected wide expansion, and the fine morphology of the silk junctions, all readily falsify the hypothesis that the regularly spaced lines of *Althepus* and *Ochyrocera* are produced by the evenly separated aciniform spigots in a single stroke.

We found that *Ochyrocera* sp. 1 build U‐modules as individual units during early web construction, then appending more modules, either below the previous ones, or in the same plane. The spiders also use similar modules to fill in flat structures, as in the dome and the lower platform. Even the filling between the upper scaffolds is made of parallel lines. We interpret that these spiders have a highly stereotyped behavioral module for filling in surfaces using parallel lines, and that this modular behavior is expressed in different contexts within the same web. Eberhard (2018, 2020) advocated the significance of modules for the evolution of web diversity by the change of context of modules during evolution. *Ochyrocera* is a special case where the same behavioral modules are used in different contexts in the same web.

What could be the cues that *Ochyrocera* uses to make parallel lines? Because these lines do not follow a strictly stereotyped motif (e.g., they usually describe a ‘U’, but also straight bands), we find more likely that the spider senses one or more of the previous lines to make a new line reproducing the contour, as orb weavers do (see Eberhard, 2020). A second complication for this would be the areas where two bands of parallel lines cross each other, because whatever the clues are, there is potential for interference. The anapid *Tasmanapis strahan*, which builds superimposed orb webs (Lopardo et al., 2011: figure 18a), would face a similar challenge. One possibility is that the crossing bands were built as modules slightly separated, one below the other. Given the highly regular modules produced by *Ochyrocera* (and probably *Althepus* as well), and their great phylogenetic distance from the orb weavers, their behaviors are worth further investigation. Because of their large size and high abundance, the Southeast Asian species of the genus *Althepus* are promising for these studies.

What are the implications for prey retention? Several areas of the silk samples of *Althepus maechamensis* were adhered to the glass; this is common for sticky webs with viscid droplets, but not for non‐sticky webs. We noticed that the parallel lines had regularly spaced thickenings, compatible with dried droplets of viscid silk, as they occur in araneoids and pholcids (see Wolff & Gorb, 2016, 2017). Moreover, the cemented intersections discussed above are rather smooth, as they occur in the intersections of sticky spirals with radii in araneids, in contrast with the fibrous attachments between dry fibers (Kullmann, 1975). We found that each anterior lateral spinneret of *Althepus maechamensis* has two piriform gland spigots with broadened shafts, very similar to those of the aggregate gland spigots of araneids, or the modified piriform gland spigots of pholcids, which are known to produce viscid silk. All this evidence suggests that *Althepus maechamensis* produces viscid sticky silk; probably, the regularly spaced parallel lines are sticky lines aimed for prey retention. It is not clear whether *Ochyrocera* sp. 1 may produce viscid silk; we could not examine the silk of *Ochyrocera* sp. 1 with SEM because the very thin lines broke in the chamber, probably burnt by the electron beam. The spinnerets of all species of *Ochyrocera* examined thus far lack spigots with broadened shafts.

Geometrically regular webs and web elements are widespread across the tree of life, and can be of different organization with rather simple webs in mesothelids and segestriids or more elaborate, complex with regular parallel web elements as in orb weavers. Our compilation of lineages with regular patterns in webs, including the radii and spirals in orb webs, the parallel lines reported here, and many other geometrical designs, shows that geometrically regular webs occur across many clades of spiders. Since many of these regular web designs have little in common in final architecture, and probably as well in sequence of construction, one should expect that several independent evolutionary origins are plausible. Our coarse approximation to mapping the phylogenetic distribution of two aspects of geometric regularity, the repetitive parallel lines or modules, and the angular regularity of radii, suggests that these two aspects of regularity probably appeared many times in the phylogeny of spiders, in distantly related clades. Thus far, the regularly spaced parallel lines only occur in Araneomorphae. The low phylogenetic signal estimated for these two aspects of geometric web regularity suggests that web design may be a poor indicator of relationships. This may be true for higher taxa and with a coarse level of abstraction of traits, as in the present study. We expect, however, that future studies using a denser taxon sampling and fine‐grained traits will find higher phylogenetic signal, reflecting the ability of trained arachnologists to identify many of the higher level spider taxa by their webs (see Eberhard, 2020). In addition, more sophisticated approaches to characterize and homologize geometric regularity can build on this summary and provide a better understanding of the behaviors involved and their evolution.

## 5 CONCLUSION

We documented for the first time regularly spaced web elements in two distantly related families, Leptonetidae and Telemidae, also distant from any of the classic champions of regular webs, the orb‐weaving spiders. We have provided evidence against a previously proposed mechanism to produce regularly spaced lines without the intervention of sensory clues in Ochyroceratidae and Psilodercidae. We summarized the widespread occurrence of geometrically regular web patterns, like those reported here, and remark that spider webs hide many mysteries to uncover, beyond the quest for the origin of orb webs.

## AUTHOR CONTRIBUTIONS

**Martin J. Ramirez:** Conceptualization (lead); formal analysis (lead); investigation (lead); visualization (lead); writing – original draft (lead). **Jonas O. Wolff:** Conceptualization (supporting); investigation (equal); writing – review and editing (equal). **Peter Jäger:** Investigation (supporting). **Martina Pavlek:** Investigation (supporting). **Abel Pérez‐González:** Investigation (supporting). **Ivan Magalhaes:** Investigation (supporting). **Peter Michalik:** Conceptualization (equal); investigation (supporting); writing – review and editing (equal).

## CONFLICT OF INTEREST STATEMENT

None declared.

## Supporting information


**Supporting information S1** Supplementary material 1

**Supporting information S2** Supplementary material 2

## Data Availability

The data that supports the findings of this study are available in the supplementary material of this article.
