# Supplementary material for: Geometric regularity in webs of non‐orb‐weaving spiders
Source: Ecol Evol. 2023 Mar 16;13(3):e9839. doi: 10.1002/ece3.9839 (PMC10019946; doi:10.1002/ece3.9839)
Supplement: Supplementary file 1 — Supporting information S1 Supplementary material 1 [file ECE3-13-e9839-s001.docx]

**Supplementary methods and data**

The phylogenetic tree was inferred from six traditional markers (12s, 16s, co1, histone h3, 18s, and 28s; see Table S1). Each marker was aligned with MAFFT (Katoh et al., 2019) using the L-INS-I algorithm with default parameters. A list of topological constraints was implemented for clades that were well supported in the most recent phylogenomic studies of spiders (Kallal et al. 2021; Kulkarni et al. 2021; Ramírez et al. 2020). Clades that were not included in phylogenomic analyses, or that were unstable in resolution between studies were not constrained; for example, the UDOH grade (Uloboridae, Deinopidae, and Oecobioidea), the root of the RTA clade in relation to Titanoecidae and Phyxelididae, the relationships of Nesticidae, Physoglenidae and Synotaxidae, and the internal relationships of Theridiidae (see Fig. S1).


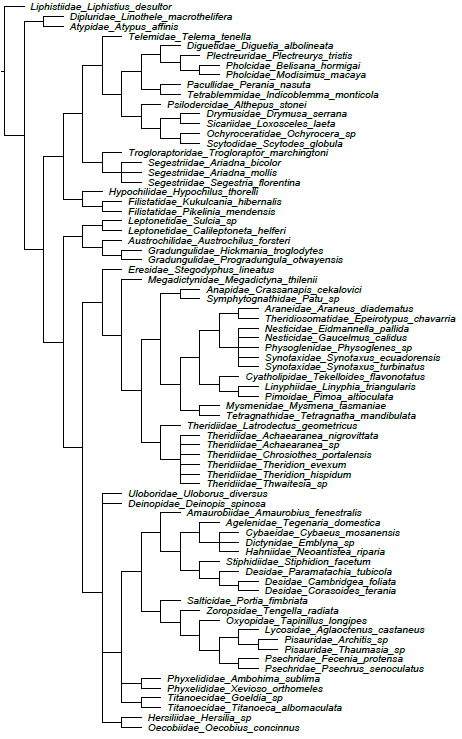


**FIGURE S1.** Tree representing the topological constraints for the phylogenetic analysis.

The sequence data were analysed with IQ-TREE 2.2.0 (Minh et al., 2020), allowing each partition to have its own substitution rate (Chernomor et al., 2016). We used the models TIM2+F+G4 (12s), GTR+F+I+G4 (16s), TIM2e+R4 (18s), GTR+F+R5 (28s), GTR+F+I+G4 (co1), and TIM2e+R4 (h3), as selected by ModelFinder (Kalyaanamoorthy et al., 2017), and calculated the maximum likelihood tree enforcing the above-mentioned topological constraints (henceforth “consensus tree”). To account for phylogenetic uncertainty, we saved 1000 ultrafast bootstrap trees (Hoang et al., 2018) for subsequent analyses.

We randomly selected 100 trees from the 1000 bootstrap sample and transformed each to an ultrametric chronogram with treePL (Smith and O’Meara, 2012) using secondary calibrations, taken from the minimum and maximum ages of the 95% highest posterior density interval of 21 dating points from the fossil-based chronogram of spiders of Magalhaes et al. (2020), as corresponding to our tree. The consensus tree was dated with the same procedure. We used a treePL smoothing parameter of 0.0001, selected by cross validation on the consensus tree, with values between 1000 and 1e-10.

| **TABLE S2.** Calibrations used to date phylogenetic trees. Tips 2 and 2 are pairs of species used to define clades. Ages are in million years before present. | | | | |
| --- | --- | --- | --- | --- |
| **Clade** | **Tip 1** | **Tip 2** | **Min. age** | **Max. age** |
| Araneae | Liphistiidae_Liphistius_desultor | Araneidae_Araneus_diadematus | 354.62 | 430.74 |
| Opistothelae | Atypidae_Atypus_affinis | Araneidae_Araneus_diadematus | 328.34 | 403.2 |
| Mygalomorphae | Atypidae_Atypus_affinis | Araneidae_Araneus_diadematus | 250.41 | 343.57 |
| Araneomorphae | Hypochilidae_Hypochilus_thorelli | Araneidae_Araneus_diadematus | 298.38 | 377.01 |
| Hypochilidae + Filistatidae | Hypochilidae_Hypochilus_thorelli | Filistatidae_Kukulcania_hibernalis | 149.26 | 311.69 |
| Synspermiata | Trogloraptoridae_Trogloraptor_marchingtoni | Pholcidae_Modisimus_macaya | 209.62 | 297.44 |
| Scytodoidea excluding Psilodercidae | Drymusidae_Drymusa_serrana | Scytodidae_Scytodes_globula | 128.33 | 214.55 |
| Pholcoidea | Tetrablemmidae_Indicoblemma_monticola | Pholcidae_Modisimus_macaya | 81.19 | 200.4 |
| Hickmania + Gradungulidae | Gradungulidae_Hickmania_troglodytes | Gradungulidae_Progradungula_otwayensis | 69.69 | 151.66 |
| Entelegynae | Eresidae_Stegodyphus_lineatus | Salticidae_Portia_fimbriata | 205.44 | 263.99 |
| Nicodamoidea + Araneoidea | Megadictynidae_Megadictyna_thilenii | Araneidae_Araneus_diadematus | 181.47 | 241.25 |
| Araneoidea | Theridiidae_Latrodectus_geometricus | Araneidae_Araneus_diadematus | 160.91 | 215.37 |
| Theridiidae | Theridiidae_Latrodectus_geometricus | Theridiidae_Theridion_hispidum | 78.74 | 159.62 |
| Oecobioidea | Oecobiidae_Oecobius_concinnus | Hersiliidae_Hersilia_sp | 117.61 | 205.09 |
| marronoids | Amaurobiidae_Amaurobius_fenestralis | Desidae_Cambridgea_foliata | 92.15 | 149.17 |
| marronoids excluding Amaurobiidae | Agelenidae_Tegenaria_domestica | Desidae_Cambridgea_foliata | 79.12 | 133.4 |
| Stiphidiidae + Desidae | Stiphidiidae_Stiphidion_facetum | Desidae_Cambridgea_foliata | 48.52 | 101.34 |
| Oval Calamistrum Clade + Dionycha | Zoropsidae_Tengella_radiata | Salticidae_Portia_fimbriata | 98.26 | 153.97 |
| Oval Calamistrum Clade | Zoropsidae_Tengella_radiata | Lycosidae_Aglaoctenus_castaneus | 92.69 | 145.33 |
| Oxyopidae to Lycosidae | Oxyopidae_Tapinillus_longipes | Lycosidae_Aglaoctenus_castaneus | 65.8 | 127.66 |
| Pisauridae + Lycosidae | Pisauridae_Architis_sp | Lycosidae_Aglaoctenus_castaneus | 33.15 | 80.21 |

The phylogenetic mapping was made using the R packages phytools, geiger, ape and sensiPhy. Two models of discrete character evolution (ER, equal rates and ARD, all rates different) were tested with Akaike information criterion using the consensus tree and the ace function of ape. Using the original tree depth (about 417 Mya), ace was not able to infer the ancestral estimations for the character “parallel” (e.g., all internal nodes with 0.5/0.5 for each state), probably because the two *Achaearanea* species, which are scored as different states, are united by a short branch, hence inferring a high rate of change (the methods fitMk and fitDiscrete of the packages phytools and geiger, respectively, also failed to infer ancestral states using the original tree depth). The tree was hence rescaled in a range of values of root depth (1, 0.5, 0.1, 0.05, 0.01, 0.001) and the ln(likelihood) values were calculated under both models (ER and ARD); the inferred ancestral states were inspected visually. For both characters, tree depths of 0.5, 0.1, 0.05, and 0.01 all produced uniform lnL values and inferred the same ancestral states. An intermediate root depth of 0.1 was used for subsequent analyses.

The AIC criterion selected the ER model in both characters, with AIC weights greater than 70% (Table S3). Both models produced similar ancestral states for each character (Fig. S2). We used the ER model for subsequent calculations.

| **TABLE S3.** Summary statistics for phylogenetic mapping on the consensus tree, using maximum likelihood. | | |
| --- | --- | --- |
|  | **parallel** | **radial** |
| lnL ER | -43.8364 | -40.1399 |
| lnL ARD | -43.7538 | -40.0998 |
| AIC ER | 89.67275 | 82.27982 |
| AIC ARD | 91.50758 | 84.19953 |
| AIC weights ER | **0.714515** | **0.723093** |
| AIC weights ARD | 0.285485 | 0.276907 |
| rates ER | 7.363635 | 6.417875 |


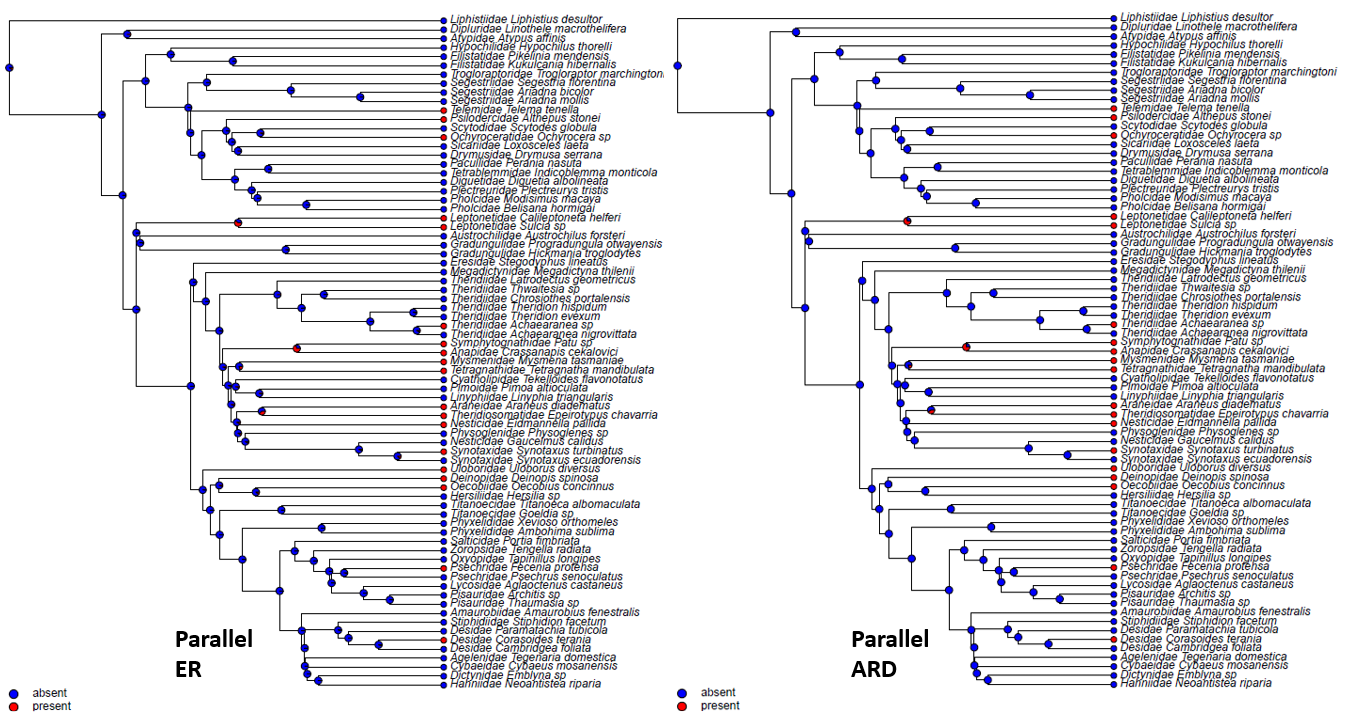


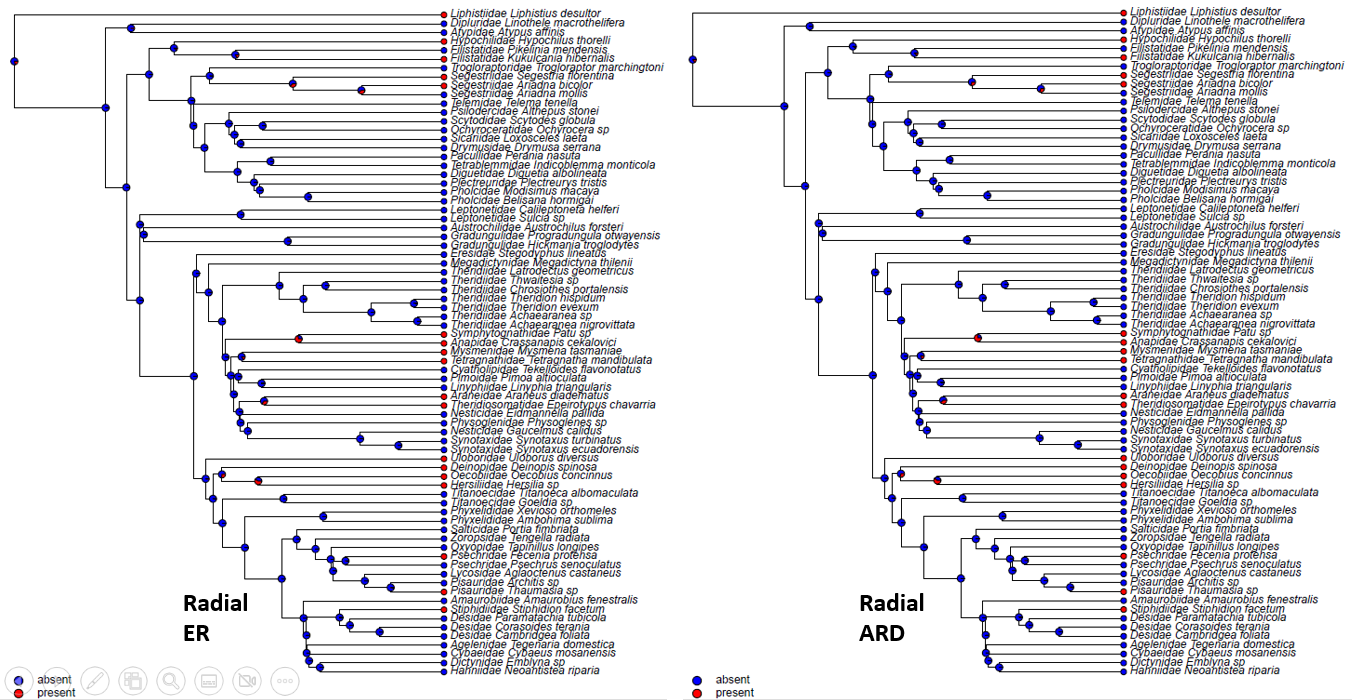


**FIGURE S2.** Character mappings under maximum likelihood, using the ER and ARD models. Both models produced similar ancestral states for each character.

The number of gains and losses over the dated consensus tree was estimated with simmaps from the package phytools, using 100 stochastic simulations (Table S3, Fig. S3). The median estimate of changes for the parallel regularity was 20 gains and 4 losses, and for the radial regularity 18 gains and 3 losses.

| **TABLE S3.** Distribution of changes from stochastic mapping on the consensus tree | | | | |
| --- | --- | --- | --- | --- |
|  | **Parallel: (1) absent, (2) present** | | **Radial: (1) absent, (2) present** | |
|  | **1→2** | **2→1** | **1→2** | **2→1** |
| min. | 11 | 0 | 12 | 0 |
| median | 20 | 4 | 18 | 3 |
| mean | 19.79 | 4.12 | 17.66 | 3.69 |
| max. | 25 | 13 | 23 | 9 |
| 95% HPD interval | [15, 24] | [0, 8] | [13, 21] | [1, 7] |


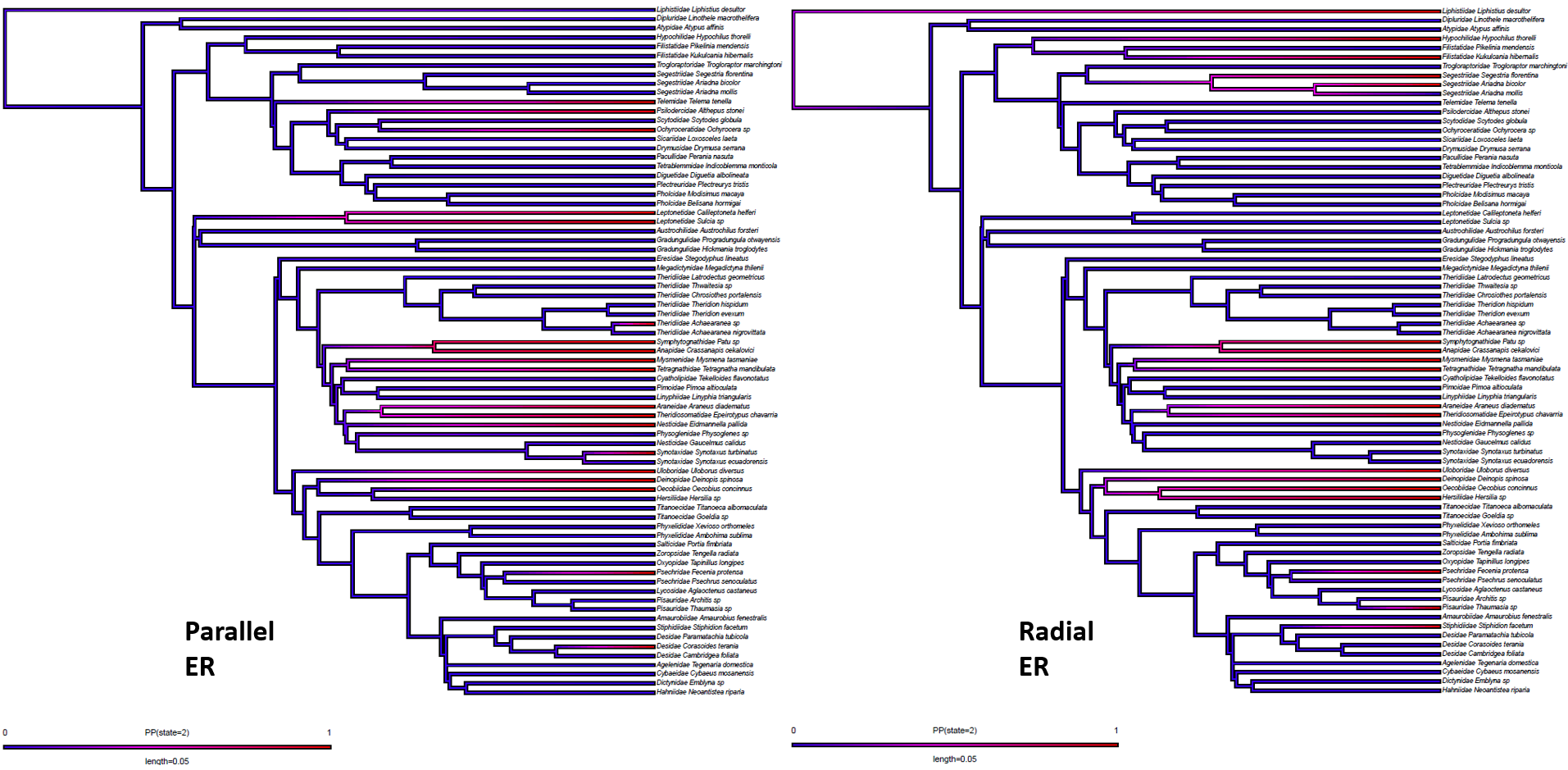


**FIGURE S3.** Stochastic character mappings using simmap.

To account for dating and topological uncertainty, 100 stochastic simulations were produced on each of 100 bootstrapped trees (each dated with treePL) (Table S4). The results are very similar to those from the consensus tree.

| **TABLE S4.** Number of gains and losses inferred from the stochastic mappings (100 simulations on each of 100 bootstrapped trees). | | |
| --- | --- | --- |
|  | mean | SD |
| parallel, gains | 19.9 | 0.33 |
| parallel, losses | 4.3 | 0.28 |
| radial, gains | 17.5 | 0.48 |
| radial, losses | 3.4 | 0.24 |

The phylogenetic mapping under parsimony was made with TNT (Goloboff and Catalano 2006). The results are summarized on Table S5, and Fig. S4.

| **TABLE S5.** Counts of transformations on the consensus tree and average over 100 bootstrapped trees | | | | |
| --- | --- | --- | --- | --- |
|  | **parallel** | | **radial** | |
|  | **consensus** | **100 trees** | **consensus** | **100 trees** |
| min. gains | 10 | 9.57 | 7 | 7 |
| max. gains | 12 | 13.35 | 13 | 12.77 |
| min. losses | 2 | 1.07 | 0 | 0.22 |
| max. losses | 4 | 4.85 | 6 | 5.99 |
| length | 14 | 14.42 | 13 | 12.99 |

**FIGURE S4.** Mapping of characters under parsimony.

The maximum likelihood and parsimony analyses are coincident in estimating, for both kinds of web regularity, a high number of independent origins and few reversals. The number of transitions is lower in the parsimony estimates, as expected. The parsimony estimations are in better agreement with current ideas of web evolution in orb weavers, i.e., in some sister groups with regular webs the ancestor is inferred regular in the parsimony mapping, but not in the maximum likelihood mapping (see for example the pairs *Epeirotypus*-*Araneus*, and *Mysmena*-*Tetragnatha* in Fig. S2 vs. Fig. S4). This effect arises from the taxon sampling reduced to a single representative per lineage, which is not problematic for parsimony, but creates problems for maximum likelihood. Take for example the sister groups *Mysmena tasmaniae* - *Tetragnatha* *mandibulata* in Fig. S2, representing the orb-weaving families Mysmenidae and Tetragnathidae. The very long branches leading to each species, combined with a short branch at the root produce the "radial" or “parallel” state inferred as convergent in both sister species. However, a denser taxon sampling of mysmenids and tetragnathids (see Kallal et al. 2020: fig. S21) shows that both families started diversifying close to their divergence, and the orb web, including its radial and parallel regularity, is ancestral for both. A denser taxon sampling and scoring of web regularities, which is out of the scope of this study, will provide a better estimation of the evolution of web regularities.
